# Intraoperative ultrasound: “Alternative eye” in lymph nodal dissection in non‐small cell lung cancer

**DOI:** 10.1111/1759-7714.14623

**Published:** 2022-10-20

**Authors:** Gaetana Messina, Mary Bove, Antonio Noro, Giorgia Opromolla, Giovanni Natale, Rosa Mirra, Francesca Capasso, Davide Gerardo Pica, Vincenzo Di Filippo, Mario Pirozzi, Marianna Caterino, Sergio Facchini, Alessia Zotta, Rita Polito, Giovanni Vicidomini, Mario Santini, Alfonso Fiorelli, Fortunato Ciardiello, Morena Fasano

**Affiliations:** ^1^ Thoracic Surgery Unit Università degli Studi della Campania “Luigi Vanvitelli” Napoli Italy; ^2^ Oncology, Department of Precision Medicine Università della Campania “L. Vanvitelli” Naples Italy; ^3^ Nutrition Science University of Foggia Foggia Italy

**Keywords:** lymph nodes, mediastinum, non–small cell lung cancer, stadiation, ultrasound

## Abstract

**Introduction:**

Staging of the mediastinum lymph nodes involvement in patients with non–small cell lung cancer (NSCLC) is an important prognostic factor determining the most appropriate multimodality treatment plan. The objective of this study is to assess ultrasound characteristics of mediastinal lymph nodes metastasis and effectiveness of intraoperative ultrasound‐guided mediastinal nodal dissection in patients with resected NSCLC.

**Materials and Methods:**

All patients undergoing video‐assisted thoracoscopic surgery lobectomy and pulmonary lymphadenectomy from November 2020 to March 2022 at the thoracic surgery department of the Vanvitelli University of Naples underwent intraoperative ultrasound‐guided mediastinal lymph nodal dissection.

**Results:**

This study evaluates whether individual B‐mode features and a compounding thereof can be used to accurately and reproducibly predict lymph node malignancy.

**Discussion:**

Intraoperative ultrasound, during systematic mediastinal lymph node dissection, is helpful in preventing lesion to mediastinal structures. Pathological nodal sonographic characteristics are round shape, short‐axis diameter, echogenicity, margin, the absence or presence of coagulation necrosis sign, and the absence or presence of central hilar structure, increased color Doppler flow, the absence or presence of calcification, and nodal conglomeration. Operating time was not substantially prolonged. The procedure is simple, safe and highly accurate.

**Conclusions:**

Ultrasonic techniques allow surgeons to detect the relationship between lymph nodes and surrounding large blood vessels during biopsy, improving the safety and simplicity of the operation, increasing the number of harvested lymph nodes, and reducing the risk of intraoperative injury; it is a fast, easily reproducible, and inexpensive method.

## Graphical abstract

Intraoperative ultrasound of mediastinal lymph nodes allows surgeons to detect metastatic lymph nodes during surgery for NSCLC. It can show the relationship between lymph nodes and surrounding structures during biopsy, improving the safety and simplicity of the operation, increasing the number of harvested lymph nodes, and reducing the risk of intraoperative injury. It is a fast, easily reproducible and inexpensive method.

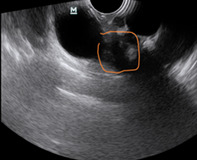

## INTRODUCTION

Lung cancer is one of the principal causes of cancer deaths worldwide. Defining the stage of the disease is crucial for planning therapy, estimating prognosis and comparison of the results. Accurate staging of the mediastinal lymph nodes involvement in patients with non–small cell lung cancer (NSCLC) is an extremely important prognostic factor and it is vital in determining the most appropriate multimodality treatment plan.^1^

Preoperative lymph nodes staging includes noninvasive strategies such as computed tomography scan (CT) and positron emission tomography (PET).^2^ It also includes invasive strategies such as endobronchial ultrasound‐guided fine‐needle aspiration (EBUS‐FNA) and endoscopic ultrasound‐guided fine‐needle aspiration (EUS‐FNA),^3^, ^4^ anterior mediastinotomy,^5^ and cervical mediastinoscopy.^6^, ^7^

However, medical imaging is unfit to provide adequate staging^8^ and the European Society of Thoracic Surgeons (ESTS) recommended a systematic nodal dissection (SND) in all cases.^9^ In a joint study from the European Respiratory Society and the American Thoracic Society, 5% to 15% of NSCLC patients classified with preoperative CT scan evaluation as stage T1N0, were found to have malignant lymph nodes by surgical dissection.^10^

SND is defined as the systematic removal of all the mediastinal tissue containing lymph nodes within anatomic landmarks, in association with intrapulmonary and hilar nodes.^11^ Whereas lobe‐specific node dissection is defined as the excision of mediastinal tissue containing specific lymph node stations depending on the lobar location of the primary tumor, based on the anatomic examination of specific lymphatic drainage patterns.^12^ The 5‐year survival depends mainly on tumor, node, metastases (TNM) status and the overall medical status of the patient. Skip transfer and occult lymph node metastasis are two theoretical bases for SND.^13^ The status of N2 lymph nodes determines the treatment strategies and prognosis of NSCLC. There is evidence that lymph node dissection improves staging, but first, it increases survival.^14^

The objective of this study is to assess ultrasound (US) characteristics of the mediastinal lymph nodes metastasis and the effectiveness of intraoperative US‐guided mediastinal nodal dissection in patients with resected NSCLC.

## MATERIALS AND METHODS

This is an observational retrospective single‐center study whose primary aim was to confirm the validity of intraoperative lung US as a safe and effective localization method for mediastinum lymph node involvement in patients with NSCLC.

All patients undergoing video‐assisted thoracoscopic surgery (VATS) lobectomy and pulmonary lymphadenectomy from November 2020 to March 2022 at the Thoracic Surgery Department of the Vanvitelli University of Naples underwent intraoperative US.

Patients underwent general anesthesia and double‐lumen endotracheal intubation with contralateral single lung ventilation. Position of the cannula was confirmed by fibroptic bronchoscopy with unilateral pulmonary ventilation. Patients were placed in the lateral decubitus position.

Triportal approach according to Hansen et al.^15^ was used. It consists of two 1–1.5 cm lower access incisions, located in the 7th or 8th intercostal space, respectively in the posterior and anterior axillary line, for two thoracoscopic ports and a 4–5 cm port incision, in the 4th intercostal space, in the anterior axillary for a utility incision.

The probe was inserted into the chest through the operating hole and mediastinal lymph node stations were explored. Mediastinal lymph node stations were subsequently labeled and their characteristics under US guidance was recorded.

**FIGURE 1:**
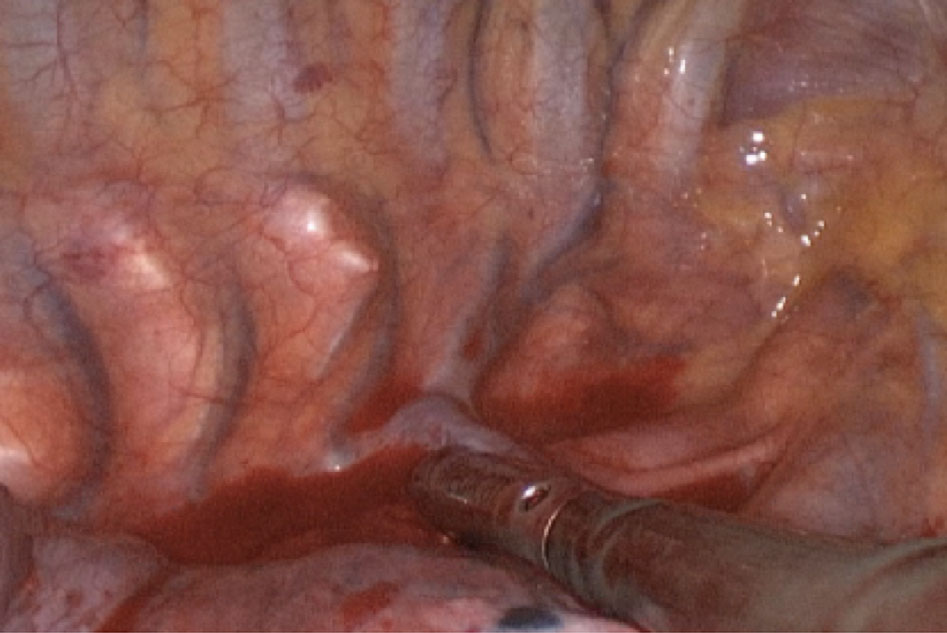
A sterile intracavitary laparoscope probe with 10 mm diameter, 38 cm length, and a flexible tip, equipped with a convex array transducer with frequencies ranging from 4 to 12 MHz, was introduced through one of the video‐assisted thoracoscopic surgery (VATS) ports.

**FIGURE 2:**
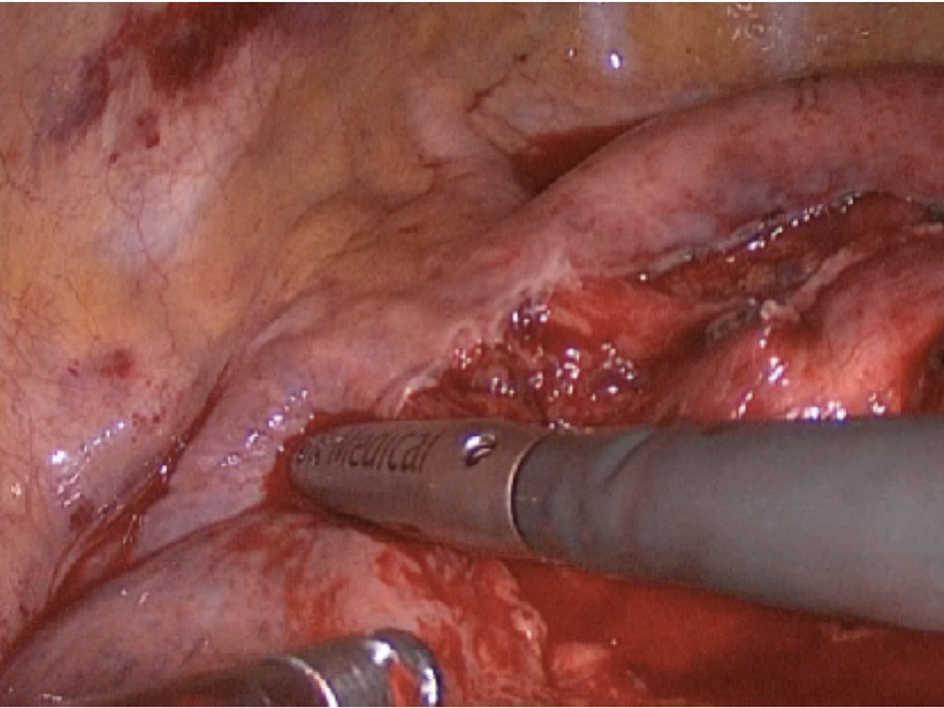
Ultrasound lymph node stations.

The US processor used for examination of lymph node stations was the BK 5000. A sterile intracavitary laparoscope probe with 10 mm diameter, 38 cm length and a flexible tip, equipped with a convex array transducer with frequencies ranging from 4 to 12 MHz, was introduced through one of the VATS ports. A setting for superficial tissue with tissue harmonics, electronic focusing at the interface level and gain <50%, were used (Figures 1, 2). The lung under examination was inflated to avoid complications because of post‐operatory pulmonary re‐expansion. By the application of pressure on the visceral pleura using the probe, a desufflation of the lung was obtained, to eliminate the air around the affected lung during the examination; the probe was positioned perpendicularly to the mediastinal tissue containing lymph nodes.

A warm sterile saline was used to improve surface contact. No change in the standardized surgical procedures was determined by the accommodation of the probe.

A 10‐minute (range, 8 e12 with 95% confidence interval [CI] [9.57–10.19]) longer operative time was required with intraoperative US compared to the surgery without intraoperative US. The lymph nodes were classified according to their sonographic characteristics of round shape, short‐axis diameter, echogenicity (heterogeneous or homogeneous), margin (indistinct or distinct), the absence or presence of coagulation necrosis sign, the absence or presence of central hilar structure, increased color Doppler flow, the absence or presence of calcification, and nodal conglomeration.

In all patients, we performed US in all lymph node stations. Subsequently on the right side, intraoperative US was performed in the upper mediastinal district from an area bounded caudally by the take‐off of the right upper lobe, superiorly by the innominate artery, anteriorly by the superior vena cava (SVC), and posteriorly by the trachea and lymph nodes with pathological nodal sonographic characteristics were removed. On the left side, intraoperative US was performed in the area between the phrenic nerve anteriorly, the vagus nerve posteriorly, up to the top of the aortic arch superiorly and caudally to the left main stem bronchus.

Lymph nodes with pathological sonographic characteristics were removed and labeled.

**FIGURE 3:**
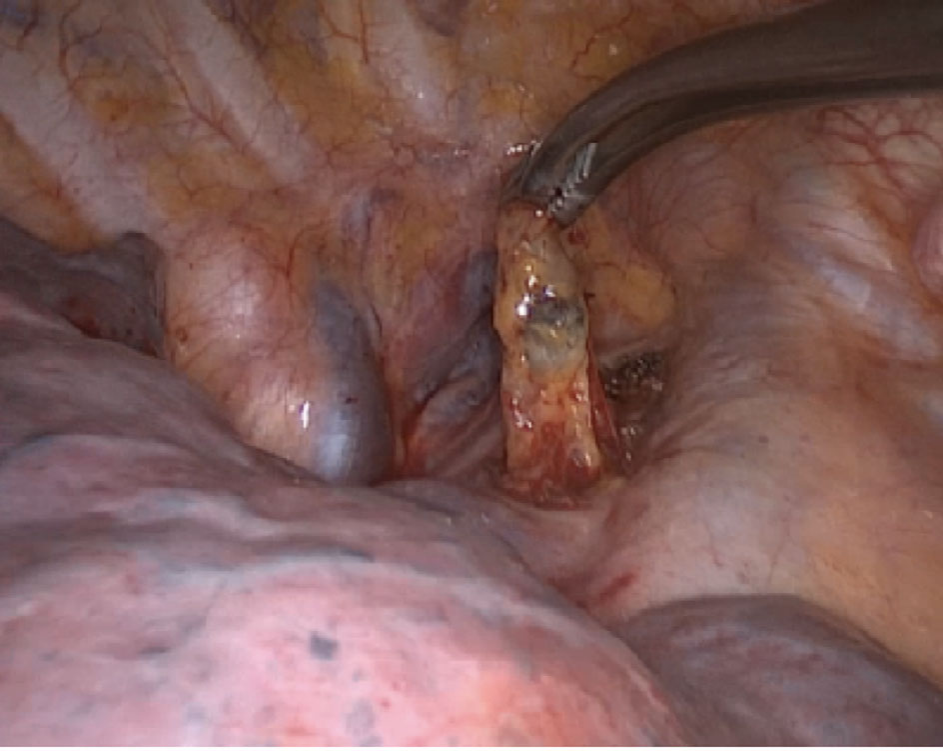
VATS lymphadenectomy under ultrasound guidance.

Next, we completed the radical lymphadenectomy and sent all the samples to pathological anatomy for histological examination. Regardless from the tumor side, complete removal of subcarinal nodes, the inferior pulmonary ligament nodes, and nodes adjacent to the caudal part of the esophagus should be accomplished. At the end of the dissection the main stem bronchi, posterior pericardium, and esophagus should be completely free of lymphatic tissue (Figure 3).

### Statistical analysis

Intraoperative US of the mediastinal lymph nodes showed: sensitivity, 86%; specificity, 95%; positive predictive value (PPV), 59%; negative predictive value (PNV), 98%; and accuracy, 95% (Table 1).

**TABLE 1:** Statistical analysis

	Sensitivity %	Specificity %	Accuracy %	Positive predictive value %	Negative predictive value %
Ultrasound	87	95	95	59	99

## RESULTS

We performed a retrospective single‐center study with 94 patients undergoing VATS lobectomy for lung cancer from November 2020 to March 2022 at the Thoracic Unit of Luigi Vanvitelli University of Naples.

All patients underwent intraoperative US of the mediastinal lymph nodes. The mean age at the time of surgery was 61 years (range, 46–81) with 95% CI (59.72–64.07). The side of lung nodules were: 27 patients (29%) right upper lobe (RUL); seven patients (7%) left lower lobe (LLL); 21 patients (22%) medium lobe (ML); 19 patients (20%) right lower lobe (RLL); and 20 patients (21%) left upper lobe (LUL).

Histology consisted of 53 adenocarcinoma (56%); two adenosquamous carcinoma (2%); 26 squamous carcinoma (24%); one pleomorphic carcinoma (1%); two colorectal lung metastasis (2%), two B‐cell lymphoma (2%), two typical carcinoid (2%), six atypical carcinoid (6%); and one inflammatory myofibroblastic tumor (1%).

Surgical resections consisted of 26 RUL (28%); six ML (6%); 21 RLL (22%); 16 LUL (17%); 19 LLL (20%); one bilobectomy (1%); three left S4 + 5 (3%); one left S6 (15); one wedge resection (1%). Operating time was prolonged for 10 (range, 8 e12) with 95% CI (9.57–10.19) minutes in all patients.

Histological examination of the lymph nodes showed six patients (6.3%) with metastatic N1 lymph nodes; five patients (5.2%) with metastatic N1–N2 lymph nodes; three patients (3.1%) with metastatic N2 lymph nodes, skip metastasis, without N1 nodal involvement (Table 2).

**TABLE 2:** Characteristics of patients

	Patients, *n* = 94
Age, years (median)	63
Sex (male), *n* (%)	60 (65%)
Side of lung nodules, *n* (%)	
RUL	27 (29%)
LLL	7 (7%)
ML	21 (22%)
RLL	19 (20%)
LUL	20 (21%)
Time to identify the lymph nodes (min) median	10 (9.57–10.19)
Histology, *n* (%)	
Adenocarcinoma	53 (56%)
Adenosquamous carcinoma	2 (2%)
Squamous carcinoma	26 (24%)
Pleomorphic carcinoma	1 (1%)
Colorectal lung metastasis	2 (2%)
B‐cell lymphoma	2 (1%)
Typical carcinoid	2 (2%)
Atypical carcinoid	6 (6%)
Inflammatory myofibroblastic tumor	1 (1%)
Surgical resections *n* (%)	
RUL	26 (28%)
ML	6 (6%)
RLL	21 (22%)
LUL	16 (17%)
LLL	19 (20%)
Bilobectomy	1 (1%)
Left S4 + 5	3 (3%)
Left S6	1 (1%)
Wedge resection	1 (1%)
TNM staging	
T1a	6 (6%)
T1b	24 (21%)
T1c	19 (20%)
T2a	23 (24%)
T2b	8 (9%)
T3	12 (13%)
T4	2 (2%)
pN	
N1	6 (6.3%)
N1 + N2	5 (5.2%)
N2 (skip lesions)	3 (3.1%)

Abbreviations: LLL, left lower lobe; LUL, left upper lobe; ML, medium lobe; RLL, right lower lobe; RUL, right upper lobe.

Pathological nodal sonographic characteristics are round shape, heterogeneous echogenicity,^16^ margin indistinct,^17^ the presence of coagulation necrosis sign,^18^ the absence of central hilar structure,^19^ increased color Doppler flow, and the presence of calcification.^20^

In total, we evaluated 573 lymph nodes ultrasonically, of which 20 were true‐positives, three were false‐negatives, and six were false‐positive, therefore, intraoperative US of the mediastinal lymph nodes showed: sensitivity, 86%; specificity, 95%; PPV, 59%; PNV, 98%; and accuracy, 95%. US‐guided mediastinal lymphadenectomy seemed to advance standard staging system. Minor complications were persistent air loss for more than 5 days in only six patients. The results show the feasibility, safety, and wide availability of US guidance for mediastinal lymph node biopsy.

## DISCUSSION

The sites of intraparenchymal lymphatic metastases, usually, are interlobar lymph nodes. Interlobar lymph nodes drain at first into the hilar nodes and after to the mediastinum.^21^ Less frequently, an atypical drainage pattern can be present; it bypasses the intrapulmonary and hilar lymph nodes and proceeds directly to the mediastinal nodes, known as skip metastases.^22^ However, mediastinal drainage tends to be lobe dependent; for this reason, the location of the primary tumor may help to detect the mediastinal lymph node stations interested.^23^, ^24^ RUL tumors, commonly metastasizes to the lower paratracheal (station 4) and pretracheal (station 3) nodes, when a single station N2 disease is present.^25^

Rarely isolated metastases from RUL tumors spread to the lower mediastinum.

In patients with multistation mediastinal disease, involvement of subcarinal (station 7) and lower mediastinal (stations 8 and 9) lymph nodes tends to be higher.

Subcarinal lymph nodes are more frequently involved in right, middle, and lower lobe tumors.

The ML tumors metastasize most frequently to the lower paratracheal nodes, whereas the lower lobe tumors drain more commonly into the lower mediastinal stations (paraesophageal and inferior pulmonary ligament). LUL tumors tend to drain mainly toward the aortopulmonary window (station 5) and the para‐aortic (station 6) lymph nodes. However, LUL tumors can metastasize to the lower mediastinal nodes and to the subcarinal nodes with a higher incidence if compared with the RUL. Tumors of the LLL drain mainly to the subcarinal lymph nodes in patients with single‐station mediastinal disease, whereas to the aortopulmonary window in multistation disease.

The lower mediastinal nodes, including paraesophageal and inferior pulmonary ligament station, have been found to be the next most usually involved mediastinal sites. The LLL, more than any other lobe, is the lobe with the highest propensity to metastasize to the contralateral mediastinal nodes.

In the last decades, anatomic studies have given an interpretation of the skipping metastatic spread, demonstrating the existence of lung lymphatic vessels that run directly to the mediastinal lymph nodes, bypassing the intrapulmonary and hilar stations. Systematic nodal dissection is determinate as the systematic removal of all the mediastinal tissue, containing lymph nodes within anatomic landmarks, in association with hilar and intrapulmonary nodes.

Different methods have been used to improve lymph node mapping, but are not completely accepted yet. Several studies used intraoperative injection of technetium‐99m sulfur colloid for sentinel node mapping, in order to improve the quality of dissection.^26^ After initial promising results, they concluded that the accuracy was worse than expected and the validation of this technique was useless.^27^

Bustos et al.^28^ performed intraoperative detection of sentinel node using Patent Blue V dye, but the black coloration of the lymph node interfered with the visualization of the dye; therefore, the identification rate was low. Menconi et al.^29^ used US color Doppler to evaluate T and N staging.

Final pathological staging is based on lymph node removal with lung resection according to various approaches (thoracotomy, robotic, and VATS) and different modalities (systematic dissection, sampling, etc.).^30^

A decisive pathway of diffusion for NSCLC is represented by mediastinal lymph nodes. The presence of N2 and N3 disease is one of the most important factors influencing prognosis and therapeutic strategies.^31^

Only one article concerning the use of intraoperative US in the detection of pathological lymph nodes has been reported in literature by Juricic et al.^22^ They used intraoperative US for mediastinal lymphadenectomy in NSCLC surgery in thoracotomy. We performed intraoperative US in VATS with a laparoscopic probe. This study evaluates US individual B‐mode features and uses US compounding to predict lymph node malignancy in a reproducible and accurate way.

**FIGURE 4:**
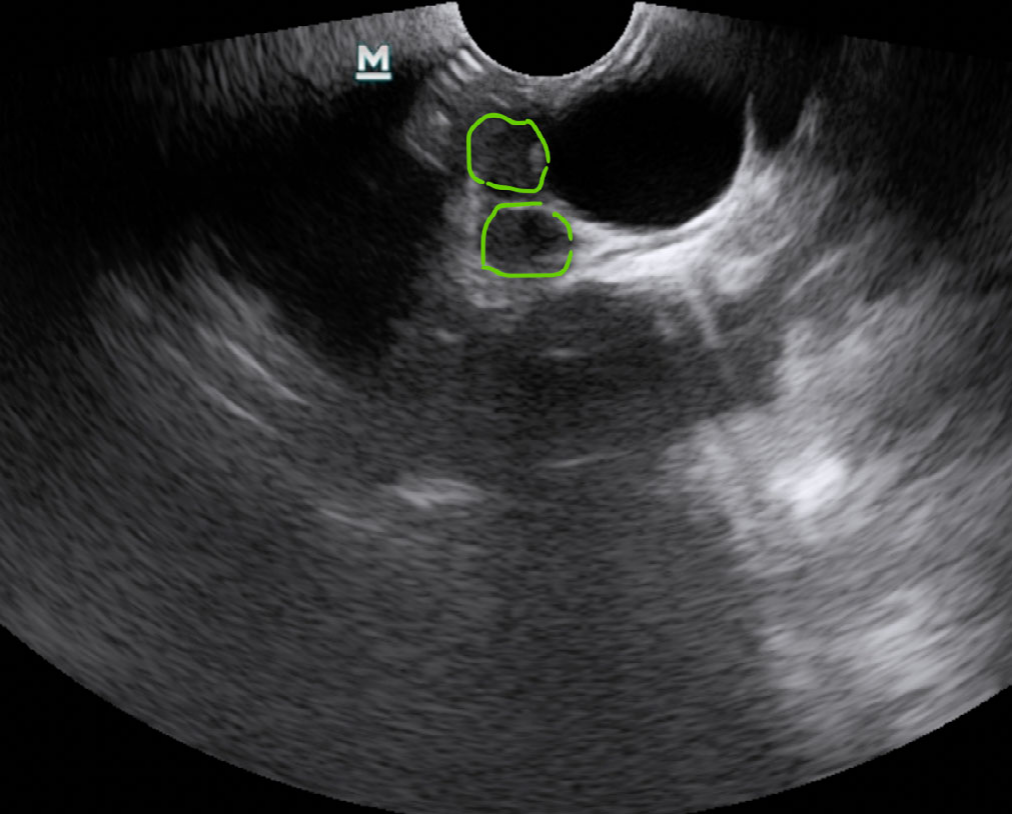
Two pathological lymph nodes ultrasonographically.

**FIGURE 5:**
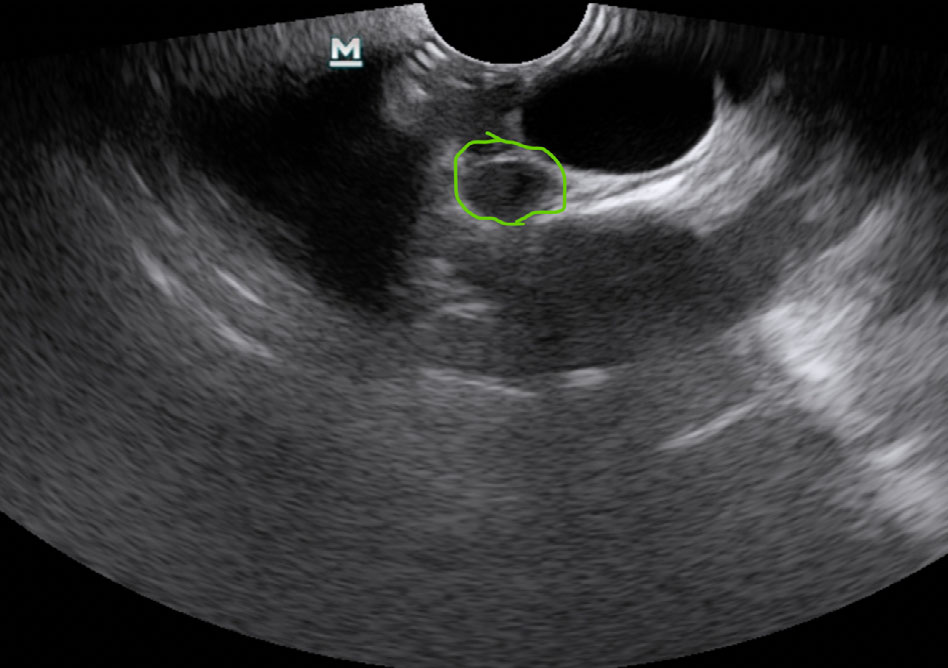
Pathological nodal sonographic characteristics round shape, the absence or presence of central hilar structure, increased color Doppler flow.

Pathological nodal sonographic characteristics are round shape, short‐axis diameter,^32^ echogenicity (heterogeneous or homogeneous),^16^ margin (indistinct or distinct),^17^ the absence or presence of coagulation necrosis sign^18^ and the absence or presence of central hilar structure,^19^ increased color Doppler flow, the absence or presence of calcification, and nodal conglomeration^20^ (Figures 4, 5).

**FIGURE 6:**
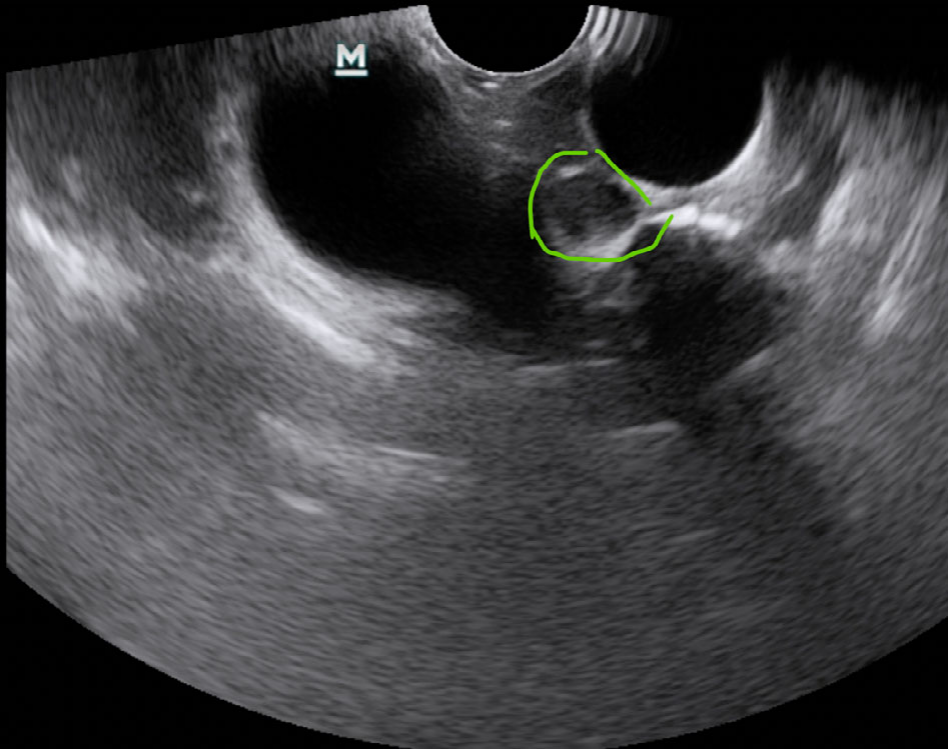
Pathological nodal sonographic characteristics.

Nodal margin was considered “distinct” when > 50% of the margin was sharply defined with a echoic border; a plain, linear, hyperechoic area in the center of the lymph node was defined as central hilar structure; a hypoechoic area without blood flow was defined as coagulation necrosis sign; the presence of multiple low echoic spots within the lymph node was defined as heterogeneous echogenicity; the presence of multiple hyperechoic punctuate areas within the lymph node was defined calcification; more than one lymph node visualized at a single lymph node station was defined nodal conglomeration. (Figure 6) Currently, the best‐made objective traditional B‐mode feature is short axis size. In line with routine clinical use, it is of value in the first identification and estimation of lymph nodes at risk of malignancy.^33^ We find that US short axis size and the absence of central hilar structure are the most objective US features for predicting lymph node malignancy.

After US evaluation, the surgical specimen suspected for metastasis was labeled with a thread of vicryl and it was sent to the pathologist. The entire lymph node was fixed in formalin and embedded in paraffin. The histological preparations was analyzed by two senior pathologists who did not know the intraoperative US data of the excised lymph nodes, and a third was consulted when there was disagreement; therefore, intraoperative US of the mediastinal lymph nodes compared to the histological evaluation showed: sensitivity, 86%; specificity, 95%; PPV, 59%; PNV, 98%; and accuracy, 95%.

Intraoperative US, during systematic mediastinal lymph node dissection, are helpful in preventing probable lesions to mediastinal structures. Operating time was not substantially prolonged. The procedure is simple, safe and highly accurate. A possible disadvantage is the requirement for experience in lung US and the method for US equipment is inexpensive. US‐guided systematic mediastinal lymph node dissection puts no patients in any risk but these methods seemed to bring survival benefit for some patients. Our results indicate that intraoperative US may have important staging implications.

Although lobectomy with systematic lymph node dissection is the standard surgical procedure in patients with early‐stage NSCLC; however, in the future intraoperative US can be use as “intraoperative eye" of resection lobe‐specific systematic node dissection in patients who are unfit for lobectomy and systematic lymph node dissection. In elderly patients with NSCLC physiological limitations, comorbidities and more post‐operative complications than younger patients can limit surgical elegibility. The segmentectomy with resection lobe‐specific systematic node dissection with use intraoperative US could be appropriate in cases of reduced pulmonary reserve or major comorbidities (e.g., low forced expiratory pressure in 1‐s, diffusion capacity of the lung to carbon monoxide, diabetes, bleeding risk, cardiovascular diseases, etc.) and smaller tumors (<2 cm).

## CONCLUSIONS

Identifying pathological mediastinal lymph nodes disease is of greatest importance because its presence significantly influences prognosis and therapeutic implications. Intraoperative US visualizes in real‐time the sample of the lymph nodes with B‐mode features including; margin distinctiveness, size, nodal heterogeneity, a central hilar structure, and presence or absence of a central necrosis sign.

Real‐time US intraoperative increases the accuracy and safety of mediastinal lymph node dissection. This technique allows surgeons to detect the relationship beetween lymph nodes and large blood vessels during lymphadenectomy, increasing the number of lymph nodes dissected and reducing the risk of intraoperative complications.

It is an easily reproducible, fast, and inexpensive method that surgeons can easily learn. Therefore, US B‐mode features are used to predict lymph node metastasis. However, higher number and location of mediastinal nodal stations in patients with resected NSCLC using US is suggested to be of considerable oncological significance. Hence, intraoperative US may have important staging implications. In conclusion, in our experience, US performed during VATS, has proved to be a safe method for the real‐time detection of lymph node metastasis. However, other clinical studies should be performed to improve intraoperative staging in NSCLC patients.

## AUTHOR CONTRIBUTIONS

Concept and design, Gaetana Messina, Mary Bove, Antonio Noro, Giovanni Natale; administrative support, Francesca Capasso, Morena Fasano; provision of study materials or patients, Alfonso Fiorelli, Giorgia Opromolla, Rosa Mirra, Giovanni Vicidomini, Mario Santini; collection and assembly of data, Giovanni Natale, Rita Polito, Davide Gerardo Pica, Sergio Facchini, Alessia Zotta; data analysis and interpretation, Vincenzo Di Filippo, Fortunato Ciardiello, Mario Pirozzi, Marianna Caterino; manuscript writing: all authors; final approval of manuscript: all authors.

## FUNDING INFORMATION

This work was supported by University of Campania Luigi Vanvitelli.

## CONFLICT OF INTEREST

All authors have completed the ICMJE uniform disclosure form. The authors have no conflicts of interest to declare.

## ETHICS STATEMENT

The authors are accountable for all aspects of the work in ensuring that questions related to the accuracy or integrity of any part of the work are appropriately investigated and resolved. The study was led in compliance with the principles of the Declaration of Helsinki. Written informed consent was obtained from all participants during preoperative communication and the protocol was approved by the Ethics Committee of the University of “Luigi Vanvitelli” of Naples (32 655/2021).
